# Cannabis in the management of PTSD: a systematic review

**DOI:** 10.3934/Neuroscience.2021022

**Published:** 2021-05-13

**Authors:** Yasir Rehman, Amreen Saini, Sarina Huang, Emma Sood, Ravneet Gill, Sezgi Yanikomeroglu

**Affiliations:** 1 Health Research Methodology, McMaster University, Hamilton, Ontario, Canada; 2 Michael DeGroote Institute of Pain and Research Center, McMaster University, Hamilton, Ontario, Canada; 3 Canadian Academy of Osteopathy, Hamilton, Ontario, Canada; 4 Faculty of Science, McMaster University, Hamilton, Ontario, Canada; 5 Faculty of Health Sciences, McMaster University, Hamilton, Ontario, Canada

**Keywords:** PTSD, cannabis, THC, nabilone, symptoms reduction, functional improvement, systematic review, meta-analysis, cannabinoid

## Abstract

**Introduction:**

Existing reviews exploring cannabis effectiveness have numerous limitations including narrow search strategies. We systematically explored cannabis effects on PTSD symptoms, quality of life (QOL), and return to work (RTW). We also investigated harm outcomes such as adverse effects and dropouts due to adverse effects, inefficacy, and all-cause dropout rates.

**Methods:**

Our search in MEDLINE, EMBASE, PsycInfo, CINAHL, Web of Science, CENTRAL, and PubMed databases, yielded 1 eligible RCT and 10 observational studies (n = 4672). Risk of bias (RoB) was assessed with the Cochrane risk of bias tool and ROBINS-I.

**Results:**

Evidence from the included studies was mainly based on non-randomized studies with no comparators. Results from unpooled, high RoB studies showed that cannabis was associated with a reduction in overall PTSD symptoms and improved QOL. Dry mouth, headaches, and psychoactive effects such as agitation and euphoria were the commonly reported adverse effects. In most studies, cannabis was well tolerated, but small proportions of patients experienced a worsening of PTSD symptoms.

**Conclusion:**

Evidence in the current study primarily stems from low quality and high RoB observational studies. Further RCTs investigating cannabis effects on PTSD treatment should be conducted with larger sample sizes and explore a broader range of patient-important outcomes.

## 1. Introduction

Posttraumatic stress disorder (PTSD) results from experiencing or witnessing an emotionally traumatic event that is perceived to present a threat to the life or physical integrity of oneself or others [1],[2]. PTSD affects approximately 8 to 9% of individuals in their lifetime and is overrepresented in the veteran population [3],[4]. PTSD manifests primarily as symptoms in cognition when trauma is re-experienced through intrusive memories, flashbacks, and/or nightmares; active avoidance of external and internal reminders of the trauma; intensified mood and emotional states such as depression, anxiety, psychological instability, impulsivity, and hyperarousal; and changes in social abilities in both personal and interpersonal functioning [2],[5].

PTSD is primarily treated by various psychotherapies and non-pharmacological interventions such as cognitive-behavioral therapy [6], exposure therapy [7],[8], support therapies, biofeedback [9], and eye movement desensitization and reprocessing [10]. Additionally, various pharmacological interventions such as selective serotonin reuptake inhibitors (SSRIs) [11],[12], serotonin-norepinephrine reuptake inhibitors (SNRIs) [13], and tricyclic antidepressants [13] aim to re-establish the balance of neurotransmitters to mitigate the symptoms. In recent years, medicinal cannabis has been increasingly used in conjunction with current psychotherapies and/or pharmacological interventions as a potentially more effective way of managing PTSD [14]–[16]. Brain morphometric studies on PTSD patients have shown alterations in the activities of the amygdala (fear conditioning), prefrontal cortex (emotional regulation), and hippocampus (memory consolidation) [17], as well as dysregulation of the hypothalamic-pituitary axis [18], which is associated with abnormal levels of neurotransmitters such as norepinephrine and serotonin. Collectively, the structural and chemical changes [19] contribute to disturbances in behavioral neurology and manifest as impulsivity, sleep disruptions, nightmares, and flashbacks. Cannabidiol, an active ingredient in cannabis, increases serotonin [20] and dopamine levels in the midbrain. This results in lower stress levels and better patient coping, ultimately reducing remission rates [14],[16],[20].

Current systematic reviews [21]–[25] exploring the effectiveness of cannabis in treating PTSD patients have key limitations such as language restrictions [21]–[25], literature searches developed from few databases [21]–[25], small sample sizes, and a narrow range of outcome focuses. Most systematic reviews focus on beneficial outcomes of cannabis intervention but do not report harm outcomes. In order to understand the scope of any treatment, consideration of both its benefits and potential risks is vital so that both patients and healthcare providers can establish realistic expectations and make informed decisions [26],[27]. The objectives of this systematic review were to assess the effectiveness of cannabis on PTSD, quality of life, social function, return to work, and harm effects such as adverse effects and dropout rates, and to critically appraise the existing literature investigating the effects of cannabis in the management of PTSD.

## 2. Methods

We conducted our review in accordance with PRISMA guidelines [28], PRISMA Harm checklists [29],[30] and chapter 24 of the Cochrane handbook for reviews involving non-randomized studies [31]. The study protocol was registered with Prospero (Registration # CRD42020164025). An experienced medical librarian refined the literature search, which included studies from the inception of each database to January 2020 in MEDLINE, EMBASE, PsycInfo, CINAHL, Web of Science, Cochrane Central Register of Controlled Trials (CENTRAL), and PubMed databases.

### 2.1. Inclusion/Exclusion criteria

Our eligibility criteria included randomized controlled trials (RCTs) or observational studies that enrolled patients 18 years or older diagnosed with PTSD and compared cannabis alone or in combination with other cointerventions, excluding experimental intervention. Our outcomes of interest were severity of PTSD symptoms, quality of life, social function, return to work, harm effects such as adverse effects, as well as dropout rates due to adverse effects, inefficacy, and all-cause dropout rates.

Title and abstract screening, full-text screening, and data extraction including risk of bias were done in duplicate and independently. Any conflicts were resolved through mutual discussion or the adjudication of a third reviewer.

### 2.2. Data synthesis strategy

As we did not have sufficient randomized controlled trials (RCTs) nor prospective cohort studies, we made an amendment in our Prospero protocol to include case control studies and case series. From the eligible studies, we extracted data on the study design, patient demographics, intervention, risk of bias, overall PTSD symptoms, quality of life, social function, and return to work outcomes. We also obtained information on harm effects such as all-cause dropout rates and those due to inefficacy, as well as adverse effects. Risk of bias was assessed with the modified Cochrane risk of bias tool [32] and ROBINS-1 [33] for RCTs and observational studies, respectively. The studies were heterogenous based on their study design, sampling strategy, sample size, and measurement of outcomes, which precluded us from pooling as reported in our Prospero protocol.

## 3. Results

**Figure 1.**
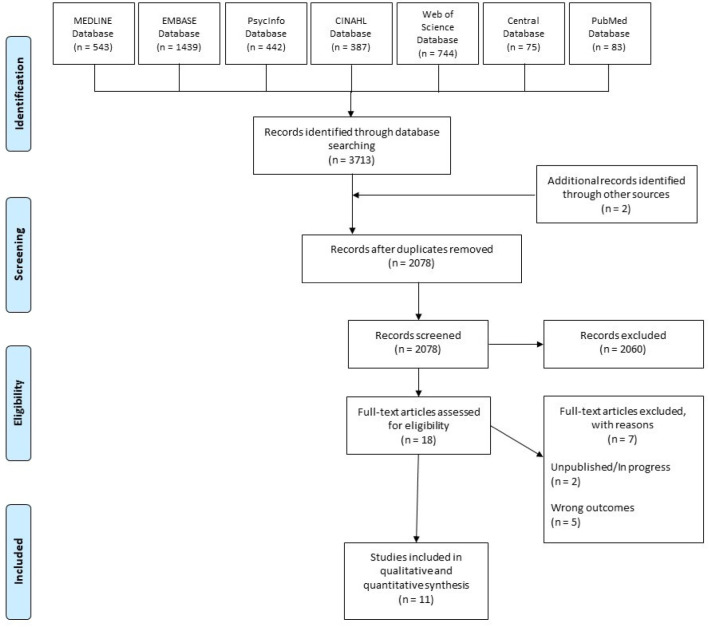


Our extensive search yielded 11 eligible studies for data extraction (Figure 1). A summary of the included studies and interventions are given in Table 1. Among the eligible studies, there were cross-over RCTs [34], chart reviews [35]–[40], cross-sectional studies [41], prospective studies [42],[43], and secondary analyses of primary RCT data [44]. The collective sample size was 4672 (range: 10 to 2276) and the median age of participants was 43.92 (range: 32.7 to 52.3). Most studies employed multiple varieties of cannabis, except for two studies that only employed nabilone [34],[39], one study which exclusively administered tetrahydrocannabinol (THC) [42], and another study which only used cannabidiol (CBD) [38]. Co-interventions for comorbid illness were permitted in six studies [34],[37]–[39],[42],[44].

As reported in our included studies, cannabis was administered as nabilone tablets [34], CBD oral capsules, CBD oral liquid sprays [38], nabilone powder dissolved in water [39], or as THC dissolved in olive oil [42]. However, seven studies [35]–[37],[40],[41],[43],[44] did not mention the method of cannabis administration.

### 3.1. Risk of bias (RoB)

The summary of risk of bias is given in Table 2. Jetly et al.'s study [34] was low risk as it met all criteria for low RoB except for the blinding of outcome assessors. All observational studies were considered high risk and none of the eligible studies met all criteria for low RoB. The most important RoB concerns were due to sampling strategies and outcomes measures.

Participant recruitment was variable among the included studies. In three studies [35]–[37], participants were invited using online surveys, where PTSD symptoms and quality of life outcomes were measured using questionnaires [35]–[37] with poor or not established psychometric properties and had very low follow-up rates. In other studies, patients were recruited from veteran populations [37],[41],[43], correctional facilities [39], outpatient clinics [38], and medical cannabis management programs for PTSD [40], or secondary data about them were extracted from previous RCTs [44]. In two studies [40],[41], non-probability sampling was employed. In Elms et al.'s study [38], patients were recruited from integrative medicine and psychiatry outpatients' clinics. These patients [38] generally avoided taking prescribed psychiatric drugs due to prior personal beliefs favoring the use of cannabis, which could have possibly augmented the placebo effect. Cameron et al. [39] had a non-representative sample from a correctional facility. It was observed that study participants would avoid criminal penalties in order to acquire cannabis, thus, the authors identified this incentive for compliance as a secondary gain from study enrollment [39]. In Ruglass et al.'s study [44], only patients who received cognitive behavioral therapy (CBT) from the authors' previous RCTs were recruited for post hoc analysis, thus, the sample was not initially intended to specifically investigate the effects of cannabis use in PTSD patients. Additionally, prior receipt of CBT may have confounded the effect of cannabis in PTSD patients [44]. Moreover, the three studies [35]–[37] which measured PTSD symptoms and quality of life, did questionnaires with poor or not established psychometric properties and had a very low follow-up rate.

### 3.2. Effectiveness outcomes

We categorized effectiveness outcomes into overall reduction in PTSD symptoms, QOL, disability, social functions, and RTW.

The summaries of effectiveness outcomes are reported in Table 3. A single RCT [34] showed that nabilone was significantly associated with a reduction in overall PTSD symptoms. Four observational studies [36],[37],[40],[43] reported that cannabis significantly reduced PTSD symptoms, whereas one observational study [41] reported an insignificant effect of cannabis on PTSD symptoms. In two studies [36],[43], cannabis use exacerbated PTSD symptoms.

Three observational studies [35],[37],[39] reported that cannabis significantly improved functional outcomes such as social function, family function, and quality of life. Only one study [43] reported the effect of cannabis on return to work. According to Wilkinson et al. [43], cannabis did not have a significant effect on return to work in PTSD patients.

### 3.3. Tolerability outcomes

We categorized tolerability outcomes into adverse effects, all-cause dropouts, dropouts due to inefficacy of cannabis, and dropouts due to adverse effects. A summary of tolerability outcomes is reported in Table 4. Overall, patient tolerability of cannabis was not explicitly reported in the included studies. Four studies [37],[40],[41],[43] did not report adverse effects nor dropout rates. In three studies [35],[36],[44], authors reported adverse effects but did not report if there were any dropout rates. The overall tolerability was variable among the included studies and varied from mild adverse effects [34],[35],[38],[42] to a worsening of symptoms [36],[43]. In Cameron et al.'s study [39], two patients developed psychosis, but one patient resumed cannabis use with no recurrence of psychosis; and other patients with existing psychosis remained stable with the antipsychotic medications.

### 3.4. Adverse effects

The most common adverse effects were dry mouth [34],[35],[39],[42] and headaches [34],[35],[39],[42], followed by behavioral changes such as psychoactive agitation [35],[39] and euphoria [35],[39]. In two studies, adverse effects were mild and did not result in any serious consequences nor complications [38],[39].

### 3.5. All-cause dropouts

Two studies [34],[39] reported all-cause dropout rates. In Cameron et al.'s study [39], 20 (19%) patients dropped from the study; and in Jetly et al.'s study [34], one patient dropped out from the placebo group prior to the cross-over.

### 3.6. Dropouts due to adverse effects and inefficacy

There were no dropouts recorded in Elms et al.'s [38] and Jetly et al.'s [34] studies explicitly due to adverse effects. Cameron et al.'s study [39] reported dropouts because of adverse effects associated with cannabis use (n = 10; 9.6%); and one patient dropped out due to inefficacy of cannabis. Roitman et al.'s [42] study reported 0 dropouts, and no other studies reported dropouts.

## 4. Discussion

In this review, we explored both efficacy and harm outcomes such as adverse effects, dropouts due to inefficacy, adverse effects, and all-cause dropout rates associated with cannabis use in PTSD patients. A cross-over RCT [34] showed that patients using nabilone had a significant reduction in overall PTSD symptoms. Its sample size was very small and only recruited five participants in each arm [34]. Data from observational studies demonstrated significant reduction in overall PTSD symptoms and improvement in functional outcomes such as quality of life, social function, and family function. Johnson et al.'s study [41] showed that cannabis use had no significant effect on overall PTSD symptoms, which may be attributed to its cross-sectional design, as temporal association could not be assessed. Only one study [43] explored the effect of cannabis on return to work but found that the effect was not significant. In most studies, cannabis was well tolerated without serious adverse effects or complications, although in two studies [36],[43], a small proportion of patients experienced a worsening of symptoms. The most common adverse effects reported in our review were dry mouth, headaches, psychoactive euphoria and agitation, and palpitations. Only one study [39] explicitly reported dropouts due to adverse effects, inefficacy, and all-cause dropout rates associated with cannabis.

**Table 1.** Baseline characteristics of the included studies.

First Author, Publication Year	Study Type	Study Period (Days)	Mean Age (SD) in Years	Participants at baseline (n)	Participants Analyzed at FUP (n)	Gender (% Females)	Cannabis Intervention	Comparator	Permitted Co-Interventions
Cameron, 2014	Retrospective chart review	1 to 252	32.7 (range: 19 to 55)	104	84	0	Nabilone; powdered form; final dosage range: 0.5–6.0 mg	N/A	Antipsychotics, sedative/hypnotics, antidepressants, antiadrenergic, NSAIDs, Acetaminophen, Opioids, Anticonvulsants, cyclobenzaprine, prednisone
Chan, 2017	Prospective (survey)	639	43.25 (range: 19 to 70)	588	540	22.28	Various varieties of cannabis; same provider	N/A	N/A
Drost, 2017	Prospective (survey)	120	N/A	647	Not clear	N/A	Various varieties of cannabis; Indica-dominant or leaning, Sativa dominant or leaning	NA	N/A
Elms, 2019	Retrospective (case series)	56	39.91 (17.39)	21	11	73	Cannabidiol (CBD); oral capsule or liquid spray; mean total dosage: 33.18 mg-48.64 mg	N/A	Anticonvulsant, antidepressant, antipsychotic, anxiolytic/sedative, beta-blocker; dietary changes, herbal supplementation, neurofeedback, and intravenous infusions of vitamins and minerals
Greer, 2014	Retrospective (chart review)	N/A	N/A	80	80	N/A	Various varieties of cannabis	No-cannabis control	N/A
Jetly, 2015	RCT (cross-over)	49	43.6 (8.2)	10	9	0	Nabilone; Cesamet & Valeant Canada tablets; dosage range: 0.5–3.0 mg	Matching placebo; Waitlist control	Antidepressants
Johnson, 2016	cross-sectional	N/A	47.1	700	700	9	Various varieties of cannabis	Case-matched non-users' control	N/A
Roitman, 2014	Prospective (open-label, preliminary)	21	52.3 (8.3)	10	10	30	THC; Dosage range of 2.5–5.0 mg THC in olive oil taken orally; twice daily	N/A	Psychopharmacological medications
Ruglass, 2017	Retrospective	98	Users: 41.63 (9.38), Non-Users: 44 (9.18)	136	136	52.21	Various varieties of cannabis; self-reported frequency	Non-users' control	Sertraline, riboflavin (for adherence)
Smith, 2017	Retrospective (chart audit)	N/A	43	100	100	3	Various varieties of cannabis; dosage range of <5, 5-9, 10, & 10 < grams	N/A	Medications for pain, depression, anti-psychotic, bipolar disorder, anxiety, ADHD, seizures, muscle relaxants, nightmares, sleep, and related effects, such as erectile dysfunction and nausea
Wilkinson, 2015	Prospective	112	51.7 (8.6)	2276	2036	3.3	Various varieties of cannabis	Cannabis use “stoppers”, “continuing users”, “starters”, & “non-users”	N/A

**Table 2.** Risk of Bias of the included studies.

Randomized studies							
Last name of first author, year			Random sequence generation	Allocation concealment	Blinding of participants	Blinding of data outcome/collector	Loss of follow-up
Jetly, 2015			Low risk	Low risk	Low risk	High risk	Low risk

Observational Studies							
Last name of first author, year	Confounding	Selection of participants into study	Classification of interventions	Deviations-intended interventions	Missing data	Measurement of outcome	Selection of reported results
Cameron, 2014	High	Low	High	Low	High	High	Low
Chan, 2017	High	Low	High	High	High	High	Low
Drost, 2017	High	Low	High	High	High	High	Low
Elms, 2019	Low	Low	Low	Low	High	High	Low
Greer, 2014	Low	High	High	Low	Low	High	Low
Johnson, 2016	Low	Low	Low	Low	Low	High	Low
Roitman, 2014	Low	High	Low	Low	Low	High	Low
Ruglass, 2017	Low	Low	Low	Low	Low	High	Low
Smith, 2017	High	Low	High	High	High	High	Low
Wilkinson, 2015	Low	High	Low	Low	High	High	Low

**Table 3.** Descriptive reporting of outcomes: PTSD symptoms.

Author, Publication Year	Scale	Intervention	Sample Size Analyzed	Authors' Conclusions
PTSD Symptoms
Cameron 2014	PCL-C	Nabilone;	104	Cannabis was associated with significant improvement in overall PTSD symptoms (P = 0.001). Pretreatment score improved from mean (SD) = 54.7 (13.0) to post intervention mean (SD) = 38.8 (7.1); P = 0.001)
Drost, 2017	Self-developed questionnaire	Cannabis (mixed)	171	77.2% of the patients, had a reduction in PTSD symptoms with the cannabis use (P = 0.0031); whereas 10.5% had not changes in PTSD symptoms.
Elms 2019	PCL-5	Cannabidiol (CBD)	11	CBD used associated with significant reduction in PTSD symptoms. At 4 weeks follow-up, 10 patients had significant reduction in overall PTSD symptoms [40.73 (12.92)]; whereas in patients symptoms worsened from baseline [PCL-5 = 63)]. At 8 weeks follow-up, 8 patients had further decreased in PTSD symptoms; whereas in three patient's PTSD symptoms worsened from four weeks follow-up.
Greer, 2014	CAPS	Cannabis (mixed)	80	Cannabis was associated with reduction in CAPS score at follow-up 22.5 (16.9); as compared to control group 98.8 (17.6); P = 0.0001). >75% reduction in CAPS score was noted with Cannabis use.
Jetly, 2015	CAPS	Nabilone	Nabilone (n = 5); Placebo (n = 4);	Nabilone was associated with reduction in overall PTSD symptoms; Nabilone = −3.6 (2.4); placebo = −1 (2.1); P = 0.03)
Johnson, 2016	PCL-C	Cannabis (mixed)	Cannabis (n = 350); control (n = 350)	No significant association between cannabis use and PTSD symptoms; Users = 59.2 (10); controls = 59.1 (11.2); P = 0.91
Roitman 2014	CAPS	THC	10	Cannabis use was not associated in reduction in PTSD symptoms (P => 0.1).
Ruglass 2017	CAPS	Cannabis (mixed)	136	No significant association between PTSD symptoms and Cannabis use was found (P > 0.30).
Smith, 2017	Survey questionnaire	Cannabis (mixed)	100	Medical cannabis uses reduced PTSD symptoms (Effect size = 1.5; P = 0.0001)
Wilkinson, 2015	Symptom severity	Cannabis (mixed)	Never used (n = 767); stoppers (n = 263); Continued users (n = 296); started (n = 738)	Cannabis was associated with worsening of PTSD symptoms. The mean for patients who continued using cannabis 38.9 (0.383) * or started cannabis 39.67 (0.226) * had higher PTSD symptoms and as compared to never users 37.71 (0.228) * and stoppers 36.64 (0.383) * respectively; P = 0.0001)
Functional Outcomes (quality of life, disability, and social functions)				
Cameron, 2014	GAF	Nabilone	103	Cannabis was associated with significant improvement in GAF (P = 0.001). Pretreatment score improved from mean (SD) = 45 (6.9) to post intervention mean (SD) = 58.2 (8.4); P = 0.001)
Chan, 2017	QOL indicators	Cannabis (mixed)	39	Medical cannabis significantly uses improvement in the overall quality of life (P = 0.03); however, individual scores on mobility, dress/ shower and activities of daily living were not significant.
Smith, 2017	Survey questionnaire	Cannabis (mixed)	100	Medical cannabis use had significant improvement in social and family life such as marital/relationship, relationships with siblings and parent children Effect size = 1.2; P = 0.0001).
Work-related Outcomes				
Wilkinson, 2015	RTW	Cannabis	Never used (n = 767); stoppers (n = 263); Continued users (n = 296); started (n = 738)	Cannabis was associated with worsening of PTSD symptoms. The mean for patients who continued using cannabis 0.594 (0.011) * or started cannabis 0.577 (0.007) * had higher PTSD symptoms and as compared to never users 0.578 (0.007) * and stoppers 0.575 (0.011) * respectively; P = 0.57)

*SE = Standard error; SD = standard deviation'

GAF = Global assessment of function; CAPS = Clinician Administered Posttraumatic Scale; PCL= PTSD check list

THC =Tetrahydrocannabinol; CBD = Cannabidiol

**Table 4.** Adverse effects and Dropout rates from the included studies.

Author	Tolerability	Adverse Effects (AE) Reported	Dropout Rate n (%)
Cameron, 2014	Two patients had psychotic episode, but one was able to restart with no recurrence. Patients with pre-existing psychosis remained with routine anti-psychotic medications.	Psychosis, sedation, dry mouth, feeling “stoned”, orthostatic hypotension, agitation, headache	N = 31 (29.8%) reported adverse effects. N = 20 (19%) withdrew from the trial; n = 10 (9.6%) withdrew from the trial due to AE; n = 4 abuse of other medications; n = 2 residential facility did not allow cannabis use; n = 2 did not want to continue; n = 1 due to inefficacy; n = 1 had no coverage.
Chan, 2017	Most patients had mild to moderate AE	Dry mouth, Psycho-active effects (feeling “high”), Sleepiness, Red/irritated eyes, Heart palpitations, Decreased memory	NR
Drost, 2017	12.3% patients had deterioration in PTSD symptoms.	Depression, anxiety, sleep problems, pain	NR
Elms, 2019	CBD was tolerated well and not patient discontinued to AE related to CBD	Daytime fogginess, gastrointestinal bloating/pain	N = 10 (48%) withdrew from the trial; authors stated reasons were largely unknown
Jetly, 2015	Cannabis was tolerated well in both arms. Patients experienced mild AE were >50% in both arms	Dry mouth, headache	N = 1 (10%) in the placebo group prior to cross over but no patient dropped out due to AE
Roitman, 2014	Four patients developed AE. These effects were mild and continued throughout the 3 weeks of treatment	Dry mouth, headache, tremor	0
Ruglass, 2017	No AE occurred	AE did not occur at the end of the study	NR

Our review possesses several key strengths. To begin, we maintained a broad search strategy as we sought eligible studies from multiple databases and did not have any language restrictions. Secondly, we focused on both patient-important outcomes such as PTSD symptoms, and functional outcomes such as social function, quality of life, and return to work, as well as harm outcomes such as adverse effects, dropout rates due to inefficacy and adverse effects, and all-cause dropout rates. However, our review also had several limitations. Firstly, we inherited the limitations of the individual studies such as small sample sizes and high risk of bias. Further, the quality of included studies was low due to high risk of bias, as none of the included studies met all criteria for low risk of bias. Moreover, one of the major limitations of the included studies was variation in sampling strategies such as the use of non-probability sampling and recruitment of patients unrepresentative of the PTSD population, such as patients with prior personal beliefs favoring cannabis use [38], or patients with secondary gain from participation, such as avoiding criminal penalty for cannabis use upon study enrollment [40]. These variations reduce the generalizability of our findings due to selection bias. Three studies [35]–[37] assessed outcomes using measures that were not previously validated, which increases risk for the measurement bias and may provide unreliable trends of PTSD symptom reduction and improvement in quality of life. Finally, most studies were single-arm observational studies and had no comparators. This also leaves us with an unanswered question concerning the relative effectiveness of cannabis with a placebo or comparator. Due to high risk of bias and heterogeneity among the included studies, we could not compare the effectiveness of cannabis with the control group; therefore, results were presented descriptively.

### 4.1. Clinical implications

Our review is unique from other systematic reviews, as previous systematic reviews [21]–[25],[45] had narrow search strategies, language restrictions, and included studies with variable goals such as the predictive association of cannabis on alcohol intake or substance use in PTSD patients, or studies which investigated individual symptoms, as opposed to patient-important symptoms. By focusing on a broader scope of patient-important outcomes such as overall PTSD symptoms, quality of life, social function, and return to work, we were able to overcome this limitation. Most systematic reviews focused on benefit outcomes of cannabis intervention, while less than 10% of reviews focus on exploring harm outcomes such as tolerability and adverse effects [46],[47]. In many systematic reviews, adverse effects are rarely reported due to a lack of standardized reporting methods [30]. To understand any treatment in its entirety, knowledge of both its effectiveness and possible harms is essential so that both patients and healthcare providers can establish realistic expectations and make informed decisions [26],[27]. We also explicitly reported harms outcomes such as dropout rates due to adverse effects, dropouts due to inefficacy, all-cause dropout rates, and adverse effects.

In most included studies, cannabis was administered in combination with other pharmacological agents, in varying potencies and through different routes such as tablets, oral sprays, or in powder form. The duration of response to cannabis was also variable among the studies. Previously, inhaled cannabis had been shown to be acutely effective in reducing PTSD symptoms, and higher doses were associated with reduced anxiety and lower frequency of intrusive thoughts [48]. However, based on data from the included studies, it is unclear whether route of administration and potency played a significant role in treating PTSD patients. Future studies should investigate the long-term effectiveness of cannabis use, and the possible influence of route of administration and potency in the management of PTSD.

Cannabis has demonstrated some success in treating other psychological conditions such as depression, anxiety disorders, and autism spectrum disorders [49]. PTSD and depression share several neurochemical mechanisms; therefore, similar interventions, including SSRIs, are often used for treating depression, anxiety disorders, and PTSD. As patients suffering from PTSD experience intrusive thoughts, flashbacks, sleep disruptions, and demonstrate avoidance behavior, these symptoms not only contribute to the persistence of PTSD, but also render treatment difficult. The prolonged stressors and symptoms persistence cause derangement in the central neurobiological process, particularly in the prefrontal cortex, hippocampus, amygdala, and cingulate gyrus [50], which can lead to nightmares, sleep disruptions, and anxiety [51],[52]. These symptoms affect patients' quality of life, functional ability, psychosocial functioning, and ability to work [53]. The mechanisms through which cannabis can reduce nightmares or sleep impairments are unknown [21],[53]. It is speculated that active ingredients in cannabis, such as THC and CBD, potentiate the memory processing and endocannabinoid systems in the brain and thus, reduce sleep impairment, nightmares [53], and overall PTSD symptoms. Patients with reduced PTSD symptoms and emotional numbing may experience better quality of life, psychosocial functioning, and working ability [54]–[56].

Another important aspect of cannabis use that we could not address in our review due to lack of evidence from included studies, was the effect of cannabis on the dissociative symptoms such as depersonalization and derealization, which could only be extracted from studies that used CAPS. Researchers interested in dissociative symptoms should consider the use of CAPS, rather than less comprehensive, although quicker, tools such as PCL. Moreover, the effect of cannabis on emotional reactivity is unclear and may exacerbate emotional dysregulation [57],[58]. Although cannabis is known to dysregulate emotional reactivity and dissociative symptoms [45], patients often prefer using cannabis as a coping mechanism in response to depersonalization experienced during acute cannabis intoxication [57]. Although symptoms such as anxiety, sleep impairment, and nightmares may improve, it is likely that more complex symptoms, such as depersonalization and derealization, can worsen. Therefore, it is important for both clinicians and patients to be fully aware of both the effectiveness and harms associated with cannabis use in order to prevent potential future complications.

## 5. Conclusions

Over the last decade, PTSD has been more frequently listed as a reason for patient request of cannabis [59],[60]. However, there is a dearth of evidence examining the benefits and harms associated with cannabis use in PTSD patients. The current evidence regarding the use of cannabis to manage PTSD is limited and based on low quality evidence. Thus, our findings should be interpreted cautiously in the context of low quality evidence due to the inclusion of studies with a small sample size, non-randomized trials, and biases in sampling strategies. There are also many important unanswered questions such as the potential of addiction and psychosis in the management of PTSD [53],[61]. Based on the limited and low quality evidence, there is a need for more rigorous RCTs with larger sample sizes to explore all benefits and harm outcomes prior to commissioning cannabis for the management of PTSD [62].

More pragmatic RCTs that compare the effects of cannabis with other pharmacological agents or psychotherapies, and with longer follow-up periods, are required to determine the effectiveness of cannabis in the management of PTSD on various patient-important outcomes. However, given that the majority of eligible studies for our review were observational, we recommend the following suggestions for future investigations. This way, findings from observational studies with smaller samples, which are often more feasible, can still aid in the scientific understanding of how cannabis impacts PTSD symptoms. Although they may not be representative of the entire PTSD population individually, systematic reviews and meta-analyses can coalesce their data to make larger scale conclusions. For example, varying subpopulations such as individuals from inpatient and outpatient facilities, and veteran populations can be recruited. Another factor that must be considered during recruitment from different geographical regions is cannabis legality [45]. Legalization of cannabis is typically followed by an increased acceptance of cannabis use [45]. Additionally, researchers can increase the quality of their studies by having blind evaluators, providing assessment training, and ensuring that treatments are carried out as planned to minimize contamination [63]. Also, future studies that use interviews as a method of data collection are encouraged to employ the Structured Clinical Interview for DSM, which is the gold standard for diagnostic interviews [63]. As well, studies using self-report methods can opt for the PTSD Symptom Scale-Self Report and PTSD Diagnostic Scale [63].

It is imperative that future research explores the impact of different cannabis preparations, methods of administration, dosages, and frequencies of use in the management of PTSD. The methodology of studies must be strictly applied so conclusions can be accurately made regarding therapeutic use. For example, the type of cannabis administered must be kept consistent amongst all participants. Ultimately, although available literature provides promise for the use of cannabis in the management of PTSD, further studies of higher quality are necessary to more adequately inform clinical guidelines.

### 5.1. Key points

1) Low quality evidence, mainly from single-arm observational studies, showed that cannabis was significantly associated with a reduction in overall PTSD symptoms, improvement in quality of life and overall function, but not with return to work. 2) A single cross-over RCT showed that nabilone was significantly associated with a reduction in overall PTSD symptoms. 3) Overall, cannabis was well tolerated. Dropout rates due to adverse effects, inefficacy, and all-cause dropouts were not consistently reported among the included studies. 4) The most common adverse effects were dry mouth, headaches, and agitation. 5) As current evidence is based on low quality, single-arm observational studies with small sample sizes, more pragmatic RCTs comparing cannabis effectiveness with other pharmacological agents and psychotherapies with longer follow-up times and larger sample sizes are required to make stronger conclusions about cannabis effectiveness in PTSD management.
